# Corn Husk Phenolics Modulate Hepatic Antioxidant Response in Nile Tilapia (*Oreochromis niloticus*) Exposed to Hypoxia

**DOI:** 10.3390/molecules26206161

**Published:** 2021-10-12

**Authors:** José Andrés Galeana-López, Cynthia E. Lizárraga-Velázquez, Crisantema Hernández, Nayely Leyva-López, J. Basilio Heredia

**Affiliations:** 1 Centro de Investigación en Alimentación y Desarrollo, A.C., Av. Sábalo Cerritos S/N, Mazatlán 82112, Mexico; jose.galeana@estudiantes.ciad.mx; 2 Cátedras CONACYT-Centro de Investigación en Alimentación y Desarrollo, A.C., Av. Sábalo Cerritos S/N, Mazatlán 82112, Mexico; 3 Centro de Investigación en Alimentación y Desarrollo, A.C., Carretera a Eldorado Km. 5.5, Col. Campo El Diez, Culiacán 80110, Mexico; jbheredia@ciad.mx

**Keywords:** antioxidant enzymes, phenolic compounds, corn by-product

## Abstract

The hypoxia conditions in intensive farming systems generate oxidative stress related to oxidative damage and mortality of fish. Corn husk meal (CHM), as a source of antioxidants, might modulate the antioxidant response and prevent the damage elicited by hypoxia. This study evaluated CHM’s ability to modulate a hepatic response in Nile tilapia exposed to hypoxia. A control and a test diet supplemented with 25 g CHM/kg feed were formulated. Ninety Nile tilapias (5.09 ± 0.55 g initial weight) were fed for 36 days to evaluate growth, feed efficiency, and hepatic antioxidant response (CAT, catalase; SOD, superoxide dismutase, and GPx, glutathione peroxidase) in normal oxygen conditions (normoxia). After the feeding trial (36 days), fish were exposed to hypoxia (1.5 ± 0.2 mg/L dissolved oxygen), and the hepatic antioxidant response was determined. There was no significant effect of CHM on growth and feed efficiency. The CAT activity was significantly increased in tilapias exposed to hypoxia and fed the test diet compared to the control group exposed to hypoxia. The SOD and GPx activities were unchanged in tilapias in normoxia and hypoxia conditions. Results suggest that CHM dietary supplementation promotes the antioxidant response in Nile tilapia exposed to hypoxia through CAT modulation.

## 1. Introduction

The high demand for fish and fishery products for human consumption worldwide has forced the aquaculture industry to develop modern aquaculture practices that are dependent on intensive farming systems [1]. This might cause temporary unstable conditions, such as changes in temperature, salinity, pH, ammonia nitrogen, and dissolved oxygen concentrations, which generates a stressful physiological environment for organisms (oxidative stress) [2]. In particular, the dissolved oxygen concentration is an important water quality criterion in intensive systems. The oxygen dissolved depletion (<2.0 mg/L) causes an insufficient O_2_ supply that does not satisfy the energy demanded by the cell (hypoxia) [3], leading to reduced growth [4], suppression of the immune system, and susceptibility to contracting infectious diseases that can generate high mortality rates [5].

Oxidative stress is the uncontrolled production of reactive oxygen species (H_2_O_2_, OH^•^, O2^•-^, HO2^•-^), which causes cellular damage through the oxidation of lipids, proteins, and nucleic acids [6]. In response to oxidative stress, the cell stimulates its maintenance through its first-line defense antioxidant, made up of the enzymes superoxide dismutase (SOD), catalase (CAT), and glutathione peroxidase (GPx) [7]. However, the activity of these enzymes is limited when there is a prolonged production of reactive oxygen species (ROS) [8]. Previous studies have reported that hypoxia restrains the response of first-line defense antioxidants in freshwater fish [9,10,11]. In this regard, research has focused on finding low-cost natural antioxidants that reduce or prevent cell damage caused by hypoxia-induced oxidative stress. Plant wastes are a rich source of phenolic compounds (PCs) widely used as antioxidant additives in aquafeeds to enhance the hepatic antioxidant response and prevent its oxidative damage in fish [6,12,13]. PCs exert their antioxidant effect through the electrons/protons donation that neutralizes ROS or modulates the SOD, CAT, and GPx activities [13,14]. Corn husk is one of the main residues of the corn crop representing a rich source of PCs from the group of hydroxycinnamic acids, such as ferulic and ρ-coumaric acids, which are responsible for the antioxidant potential of corn husk [15,16].

Tilapia is a freshwater fish of commercial interest throughout the world. Tilapia production has grown rapidly [17] which intensifies its cultivation, so this species is generally exposed to oxidative stress [18]. Therefore, the development of foods supplemented with natural antioxidants for tilapia has received significant attention from the aquafeed industry [13]. In this regard, studies have demonstrated that dietary PC inclusion in banana and elderflower protects against oxidative damage, by enhancing the enzymatic antioxidant defense (SOD, CAT, and GPX) of farmed fish [19,20].

Recent studies have reported that the dietary inclusion of corn husk extract (100 and 200 mg PCs/kg feed) and corn husk meal (25 g/kg feed) as a source of PCs increase the tilapia diet antioxidant capacity, and enhance the hepatic antioxidant response of tilapia cultivated in optimal environmental conditions for the species, respectively [15,21]. However, no studies demonstrate the antioxidant effectiveness of PCs from corn husk in tilapia under oxidative stress induced by inherent conditions of intensive fish production systems, for example, in a low concentration of dissolved oxygen. Therefore, this study evaluated corn husk’s ability to modulate a hepatic antioxidant response in Nile tilapia exposed to hypoxia.

## 2. Results

### 2.1. Phenolic Compounds Profile from Corn Husk

Three hydroxycinnamic acids (chlorogenic acid, ferulic acid, and *p*-coumaric acid) and one hydroxybenzoic acid (*p*-hydroxybenzoic acid) were identified and quantified. Ferulic acid was the major phenolic compound in corn husk meal (CHM), contributing to 68.37% of the total PCs identified, followed by *p*-coumaric acid. *p*-hydroxybenzoic acid and chlorogenic acid presented lower concentrations (15.38 mg/100 g and 10.15 mg/100 g, respectively) (Table 1).

### 2.2. Growth Performance and Feed Efficiency

The weight gain (WG), specific growth rate (SGR), feed intake (FI), feed conversion ratio (FCR), hepatosomatic index (HSI), and survival (S) were not significantly affected by supplementation of CHM (test diet) in the diet of Nile tilapia (Table 2). Tilapia survival was greater than 90%, and no significant difference (*P* > 0.05) among organisms fed with control and test diets was observed.

### 2.3. Hepatic Antioxidant-Enzyme Activities

**Figure 1:**
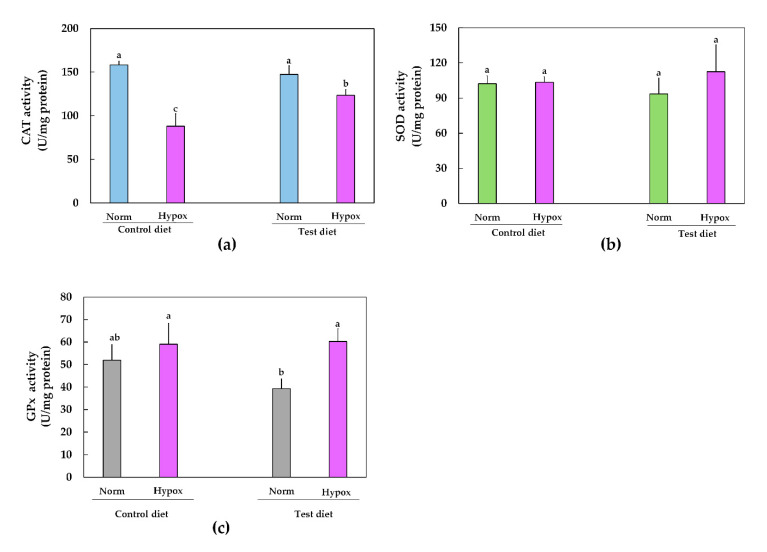
Effect of CHM (test diet) on hepatic (**a**) CAT, (**b**) SOD, and (**c**) GPx activities of Nile tilapia in normoxia (Norm) and exposed to 5 h of hypoxia (Hypox). Different letters indicate significant differences (*P* < 0.05) among treatments using two-way analyses of variance (ANOVA) followed by Tukey’s post hoc.

Exposure of organisms to hypoxia (or stress conditions) caused a significant reduction in the CAT activity. The CAT activity was lower (88.11 U/mg protein) in fish fed a control diet and exposed to hypoxia, equivalent to a 44% reduction in CAT activity. The CAT activity significantly increased in fish fed a test diet (CHM supplemented diet) compared to fish fed a control diet, with both fish exposed to hypoxia (Figure 1). Before and after hypoxia exposition, there was no statistical significance in SOD and GPx activities in fish fed control or test diets. However, the GPx activity was slightly reduced (24%) in fish fed CHM supplemented diet compared to fish fed a control diet, with both exposed to normoxia.

## 3. Discussion

The supplementation of hydroxycinnamic acids from corn husk in fish diets exerts antioxidant effects in vitro and in vivo [15,22]. This study identified ferulic (1293.405 mg/100 g) and *p*-coumaric (573.874 mg/100 g) acids as the most abundant hydroxycinnamic acids in corn husk. Vazquez-Olivo et al. [16] also reported that ferulic and *p*-coumaric acids are the main PCs in corn husk; however, they found lower values of ferulic (455.50 mg/100 g) and *p*-coumaric (351.550 mg/100 g) acids than the ones reported in our study. These differences could be influenced by environmental, pre- and post-harvest, and drying method conditions [23]. For example, we used a drying temperature of 40 °C, while Vazquez Olivo et al. [16] used a drying temperature of 70 °C. Previous reports have indicated that a high temperature can cause PC losses in plants, due to cell wall disruption and rupture, and exposure of PCs to heat, light, and oxygen during drying [24,25]. Therefore, the high concentration of hydroxycinnamic acids reported in this study is mainly attributed to the drying temperature (40 °C), which is convenient for the aquafeed industry because it reduces the production cost of CHM as an antioxidant additive in tilapia diets.

The dietary supplementation of plant extracts or powder as sources of natural antioxidants (PCs, alkaloids, terpenoids, and essential oils) could benefit or negatively affect the growth performance and feed efficiency of fish [18,26]. Hassan et al. [27] indicated that the dietary supplementation of medicinal plants, such as turmeric (10 g/kg feed), rosemary (10 g/kg feed), and thyme (10 g/kg feed), promote growth parameters in Nile tilapia. Similarly, Shourbela et al. [18] reported that using *Moringa oleifera* extract (50, 100, and 400 mg/kg feed) as a feed additive improves the growth performance in the same species through the feed intake increase and feed utilization. On the other hand, Dongmeza et al. [26] reported a considerable decrease in growth of Nile tilapia fed supplemented diets with Moringa leaf extract (10.6 and 17.7 g/kg feed). In the present study, the CHM dietary inclusion did not affect growth performance and feed efficiency in Nile tilapia. These findings agree with other studies conducted on Nile tilapia fed supplemented diets with condensed tannin isolated from grape seed (100, 200 and 400 mg/kg) [28] and orange peel fragment (2, 4, 6 and 8 g/kg feed) [13]. Based on the studies above, we could infer that the biological properties of plant antioxidants depend on the type of plant and its concentration in the tilapia feed. Therefore, in this study, we consider that PCs identified and other bioactives unidentified (fibers, lignin, and chlorophyll) in CHM and the dose of CHM used did not affect the growth of tilapia, which indicates that CHM can be used as a functional additive in tilapia diets without compromising the growth of the species.

Hypoxia reduces the mitochondrial oxygen consumption [29], rendering cells’ temporal arrest in the cell cycle and reducing energy consumption; and the increase in ROS production, mainly O_2_^•-^ and H_2_O_2_ at complex III of the mitochondrial electron transport chain [30,31]. The accelerated increase in O_2_^•-^ and H_2_O_2_ triggers oxidative stress, decreasing cellular antioxidant defenses [14], which causes oxidative liver cell damage because the liver is the main metabolizing and detoxifying organ [12]. Previous studies have reported that hypoxia-induced oxidative stress inhibits the first-line antioxidant defense, made up of CAT, SOD, and GPx in the liver of freshwater fish [9,14]. In this regard, PCs can increase the antioxidant defenses during oxidative stress via the nuclear factor derived from erythroid 2 (Nrf2) activation, inducing the transcription of the antioxidant enzymes CAT, SOD, and GPx [32,33], which maintain homeostasis of the redox state through the suppression or prevention of the ROS formation in cells to prevent oxidative damage [7,34]. First, SOD dismutate the O_2_^•-^ to H_2_O_2_ in mitochondria, cytosol, and peroxisomes, then H_2_O_2_ is reduced to H_2_O by CAT in peroxisomes and by GPx in mitochondria and cytosol [7].

Previous studies in fish farming have demonstrated that PCs can increase antioxidant defenses in fish exposed to hypoxia. For example, 6% dietary orange peel, as a source of PCs, increased the hepatic CAT, SOD, and GPx activities in Nile tilapia exposed to heat/hypoxia-induced stress (32 °C/2.3 mg/L dissolved oxygen) for three days [13]. Peng et al. [28] reported that the dietary inclusion of condensed tannins (400 mg/kg) increased the hepatic CAT activity in Nile tilapia exposed to 10 h of hypoxia (1 mg/L dissolved oxygen). The dietary inclusion of *Yucca schidigera* extract as a source of PCs increased SOD and GPx activities in liver Nile tilapia exposed to hypoxia (2.2–2.6 mg/L dissolved oxygen) [2]. In the present study, the CAT activity was reduced by 44% in tilapia fed the control diet and exposed to hypoxia, while the CAT activity was reduced by 16% in tilapias exposed to hypoxia and fed CHM supplemented diet (Figure 1a); this possibly indicates that PCs from CHM promote cell homeostasis by increasing CAT activity via Nrf2 activation. Contrarily, the hepatic SOD and GPx activities were unchanged in tilapias in normoxia and hypoxia conditions which were fed experimental diets (Figure 1b,c). This might provide information on the mechanism used by hepatic GPx and SOD in Nile tilapia, as a strategy of adaptation to the oxidative stress-induced low levels of dissolved oxygen; however, further studies on the mechanism of the hepatic antioxidant response of Nile tilapia exposed to chronic stress-induced hypoxia are needed to support our findings.

## 4. Material and Methods

### 4.1. Processing of Corn Husk

Corn husks were collected from a local market in Mazatlán, Sinaloa, México. Fresh corn husks (~30 kg) were manually washed to remove impurities, dried at 40 °C for 24 h in a forced air convection oven, and grounded to a particle size of 250 μm using a hammer mill (California Pellet Mill Laboratory Mill Champion, Waterloo, IA, USA). CHM was stored at −4 °C until use.

### 4.2. Phenolic Compound Extraction from Corn Husk

PCs from CH were extracted, according to Adom and Liu [35], with minor modifications. CHM (0.5 g) was homogenized in 80% ethanol and shaken in an Orbit Environ Shaker (Lab-Line, Melrose Park, IL, USA) at 200 rpm for 12 h at room temperature. The mixture was centrifuged (3000 g, 15 min, 4 °C), and the supernatant was collected and stored at −20 °C until analysis. The pellet was digested with 2 M sodium hydroxide at 95 °C for 30 min to extract bound PCs. Digested pellets were incubated at room temperature, shaken at 200 rpm for 1 h and neutralized with 37% HCl. Hexane was used to remove the lipids content by centrifugation. The supernatant containing hexane was discarded, and the pellet was washed five times with ethyl acetate to drag PCs. Ethyl acetate was evaporated at 35 °C using a rotavapor R-114 (Labortechnik AG, Flawil, Switzerland). Concentrated PCs were reconstituted with 2 mL of 80% ethanol. Supernatants stored were mixed and used to identify and quantify the PCs.

### 4.3. Identification and Quantification of PCs by UPLC-ESI-Q-ToF-MS/MS

PCs from CHM were identified and quantified through ultra-performance liquid chromatography (UPLC) using an ACQUITY UPLC; H-Class system (Waters, Milford, MA, USA) coupled to a G2 XS Quadrupole-Time-of-Flight (Q-Tof) mass spectrometer (Agilent, Santa Clara, CA, USA) equipped with electrospray ionization (ESI). PCs were separated at 40 °C with an ACQUITY BEH C18 column (1.7 μm, 2.1 × 50 mm). The elution solvents were 0.1% formic acid (A) and acetonitrile (B) at a flow rate of 0.3 mL/min. The gradient procedure was as follows: 0 min, 95% (A); 5 min, 70% (A); 9 min, 30% (A); 14 min, 0% (A); 14.5 min, 0% (A); 15 min, 95% (A); 16 min, 95% (A). An electrospray source in negative mode was used to collect mass spectra under the following conditions: nitrogen gas; desolvation temperature, 350 °C; desolvation gas, 13.3 L/min; capillary voltage, 1500 V; and fragmentor voltage, 30 V. PCs were quantified by calibration curve using caffeic, chlorogenic, p-coumaric, ferulic, p-hydroxybenzoic, and sinapic acids, and rutin as standards.

### 4.4. Diet Preparation

Two isonitrogenous (40% crude protein) and isolipidic (9% lipid) diets were formulated (Table 3). The control diet was formulated without the CHM addition. Based on the positive antioxidant effect of CHM (25 g/kg feed) in Nile tilapia previously reported [15], a test diet was prepared and supplemented with 25 g of CHM/kg feed. Diets were elaborated as described below: the macronutrients (fish meal, cellulose, cornstarch, and alginate) were mixed in a model AT-200 Hobart mixer (Offenburg, Germany). The micronutrients (mineral premix, vitamin premix, and vitamin C), fish oil, soy lecithin, and water were added afterward and mixed until a homogeneous mixture was obtained. CHM was added with macronutrients during test diet preparation. The resulting mash was passed through a model 22 meat grinder (Torrey®, Monterrey, México ) to produce pellets, which were dried in a forced-air oven at 37 °C for 12 h, and manually reduced to a size of approximately 2 mm. Diets were stored at 4 °C in labeled containers.

### 4.5. Chemical Analysis of Diets

Dry matter (method 4.1.06), crude protein (method 954.01), crude lipid (method 4.5.05), and ash (method 32.1.05) contents were determined following standard methods of the Association of Official Analytical Chemists [36]. Dry matter was evaluated by gravimetry using an oven at 105 °C for 12 h. Protein content was analyzed using a LECO FP-528 nitrogen analyzer (LECO Instrument Corporation St. Joseph, MI, USA). Lipid content was determined using a micro Foss Soxtec Avanti 2050 Automatic System (Foss Soxtec, Hoganäs, Sweden) using petroleum ether as solvent. Ash content was determined by calcination at 550 °C for 6 h using a muffle furnace (Fisher Scientific International, Pittsburgh, PA, USA).

The PC concentration of the experimental diets was determined following the methodology used by Galeana-López et al. [15]. The result was expressed as mg of gallic acid equivalent (g GAE)/kg feed (Table 3).

### 4.6. Feeding Trial (Experiment 1)

Nile tilapias (*Oreochromis niloticus*) used in this study were obtained from a commercial fish farm (Nayarit, Mexico). The experimental design was completely randomized and consisted of two treatments and three replicates. A group of 90 masculinized tilapias (5.09 ± 0.55 g initial body weight) were randomly distributed in 6 tanks (140 L) in groups of 15 fish per tank and submitted to normoxia. Nile tilapias were fed three times a day (8:00, 12:00 and 16:00 h) at 10% and 6% of total biomass for 14 and 22 days, respectively. The fecal matter was carefully siphoned out from the bottom of each tank twice daily, and 20% water volume was renewed daily. The water temperature was controlled at 28 ± 1.2°C, and the dissolved oxygen concentration was maintained at 5.0 ± 0.3 mg/L throughout the experiment. Before the first feed, water parameters were monitored daily using a YSI 85-10FT oximeter (YSI Inc., Yellow Springs, OH, USA). An artificial photoperiod was maintained at 12:12 h light: dark cycle.

#### 4.6.1. Growth Parameters and Feed Efficiency (Experiment 1)

All fish were collected and measured every two weeks (mean body weight) to determine biometric parameters. At the end of the feeding trial, two fish from each tank were euthanized with an overdose of clove oil (400 mg/L) and dissected to obtain liver and determine the hepatosomatic index. The dead fish were recorded and weighed during the feeding experiment to calculate the survival and the feed conversion ratio.

The growth and feed efficiency of tilapia were evaluated in terms of the following indices: WG, SGR, FI, FCR, HSI, and S. We used the following formulae:

WG (g) = final mean weight (g) − initial mean weight (g)

SGR (%/day) = 100 [((ln final weight) − (ln initial weight))/time (days)]

FI (g/fish) = Σ i70 [(total feed consumption (g))/(number of fish)/number of days]

FCR = FI (g)/weight gain (g)

HSI (%) = 100 [liver weight (g)/body weight (g)]

S (%) = 100 [(final count)/(initial count)]

#### 4.6.2. Liver Collection in Normoxia Condition (Experiment 1)

At the end of the feeding trial, two fish from each tank were randomly selected, euthanized with an overdose of clove oil, and dissected to obtain the liver and determine the antioxidant enzyme activities.

### 4.7. Hypoxia-Induced Oxidative Stress and Antioxidant Enzymes (Experiment 2)

Fifteen fish per treatment were taken from the remaining fish in the feeding trial and transferred into six tanks, with five fish/tank. Tanks were exposed to a controlled hypoxia condition (1.5 ± 0.2 mg/L dissolved oxygen) by injecting nitrogen gas into the water. During the experiment, the temperature was controlled at 28 °C ± 0.5. After 5 h of stress without feeding, two fish per tank were collected and euthanized with an overdose of clove oil, and dissected to obtain liver and measure the SOD, CAT, and GPx activities.

The antioxidant enzyme activities were determined as described below. The liver was manually homogenized on ice using 300 μL of PBS buffer (pH 7.4) and centrifuged at 3000 g for 15 min at 4 °C. The supernatant was used to determine total protein content using Bradford’s reagent [37] and bovine serum albumin as a standard, and enzyme activities were determined using Cayman Chemical kits (Ann Arbor, MI, USA). One unit of CAT was defined as the amount of enzyme that catalyzed the formation of 1 nmol of formaldehyde per minute at 25 °C. One unit of GPX was defined as the amount of enzyme that catalyzed the oxidation of 1 nmol of NADPH to NADP+ per minute at 25 °C. One unit of SOD was defined as the amount of enzyme needed to exhibit 50% dismutation of the superoxide radical. All results were expressed as specific enzyme activity in U/mg protein.

### 4.8. Statistical Analysis

Before statistical analysis, the data were tested for normality (Kolmogorov–Smirnov test) and homogeneity of variance (Levene’s test). Percent data (SGR, S, and HSI) were arcsine transformed prior to analysis, but results were reported as a percentage. One-way analyses of variance were carried to evaluate growth performance (*P* < 0.05), followed by Tukey’s multiple comparison test. The CAT, SOD, and GPx activities were evaluated using two-way analysis of variance (two independent variables: oxygen level and diet), followed by Tukey’s post hoc when significant differences were found (*P* < 0.05). Statistical analyses were carried out with Minitab version 17.1 (Minitab Inc., State College, PA, USA).

## 5. Conclusions

The CHM supplementation increased the hepatic CAT activity in Nile tilapia exposed to hypoxia without affecting SOD and GPx activities. Based on these findings, the dietary supplementation of CHM in the doses tested improves the hepatic antioxidant response of Nile tilapia exposed hypoxia-induced oxidative stress. However, further studies are necessary to fully understand the mechanisms by which CHM exerts its modulatory effect on antioxidant enzymes in fish exposed to stress.

## Figures and Tables

**Table 1:** Qualitative and quantitative characterization of phenolic compounds in corn husk by UPLC-ESI-Q-ToF-MS/MS.

Phenolic Compound	Rt * [min]	*m/z* [M − H]^−^	Fragment Ions MS/MS (*m/z*) [M − H]^−^	Molecular Formula	Concentration (mg/100 g)
Chlorogenic acid	3.73	353.08	191.05,353.08	C_16_H_18_O_9_	10.15
Ferulic acid	5.45	193.058	134.03,178.02,193.04	C_10_H_10_O_4_	1293.41
*p*-coumaric acid	5.06	163.047	117.04,163.03	C_9_H_8_O_3_	573.87
*p*-hydroxybenzoic acid	3.79	137.032	137.02,122.88,137.01	C_7_H_6_O_3_	15.38

* Rt = retention time (Supplementary Materials Files S1–S4)

**Table 2:** Growth parameters and feed efficiency of Nile tilapia fed experimental diets for 36 days.

Variable	Control Diet (without CHM)	Test Diet (25 g CHM)
IW (g)	5.09 ± 0.10	5.09 ± 0.08
FW (g)	21.40 ± 1.34	21.92 ± 1.34
WG (%)	320.50 ± 26.33	330.71 ± 24.25
SGR (%/day)	3.99 ± 0.18	4.05 ± 0.15
FI (mg/fish/day)	782.73 ± 64.43	803.79 ± 70.56
FCR	1.73 ± 0.02	1.72 ± 0.03
HSI (%)	1.92 ± 0.52	2.06 ± 0.66
S (%)	93.33 ± 6.67	95.56 ± 3.85

Values are mean ± SD for three tanks per group, fifteen fish each. There were no significant differences (*P* > 0.05) among treatments, using one-way analyses of variance (ANOVA) followed by Tukey’s post hoc. IW: initial weight, FW: final weight, WG: weight gain, SGR: specific growth rate, FI: feed intake, FCR: factor conversion ratio, HSI: hepatosomatic index, and S: survival.

**Table 3:** Ingredients, proximate composition, and PC content of the experimental diets for Nile tilapia.

Ingredient (g/kg)	Control Diet (without CHM)	Test Diet (25 g CHM)
Fish meal (sardine) ^a^	615	615
Fish oil ^b^	43	43
Cellulose ^c^	50	25
Corn husk meal ^d^	-	25
Cornstarch ^c^	261.4	261.4
Alginate ^c^	20	20
* Mineral premix ^e^	5	5
** Vitamin premix ^e^	5	5
Vitamin C ^f^	0.6	0.6
Composition (g/kg dry matter)		
Dry matter	946.45	945.96
Crude protein	449.99	449.63
Crude lipid	93.95	93.54
Ash	201.04	203.76
NFE ^g^	255.02	252.79
Phenolic content (g GAE/kg)	0.00	0.28

^a^ Selecta de Guaymas, S.A de C.V, Guaymas, Sonora México. ^b^ Proteínas marinas y agropecuarias S.A. de C.V., Guadalajara, Jalisco, México. ^c^ Droguería cosmopolita, S.A. de C.V. México, D.F., México. ^d^ Corn husk obtained from local supply center at south of Sinaloa, México. ^e^ Trout Nutrition México S.A. de C.V. (by cortesy). * Mineral premix composition: manganese, 100.00 g; magnesium, 45.00 g; zinc, 160.00 g; iron, 200.00 g; copper, 20.00 g; iodine, 5.00 g; selenium, 0.40 g; cobalt 0.60 g. ** Vitamin premix composition: vitamin A, 2400 IU/g; vitamin D3, 2250 UI/g; vitamin E, 160.00 g; vitamin K3, 8.00 g; thiamine B1, 20.00 g; riboflavin B2, 40 g; pyridoxine B6, 16.00 g; vitamin B12, 80.00 mg; pantothenic acid, 60.00 g; nicotinic Acid, 160.00 g; folic Acid, 4.00 g; biotin, 0.50 g; vitamin C, 100.00 g; choline 300.00 g, excipient 1046.85 g. ^f^ DSM Nutritional Products Mexico S.A. de C.V., El Salto, Jalisco, Mexico. ^g^ NFE (nitrogen free extract) calculated by subtraction, 1000 − (crude protein + crude lipid + ash + phenolic compounds).

## Data Availability

The data presented in this study are available in this article.

## Supplementary Materials

The following are available online. File S1: Chlorogenic acid mass spectrum; File S2: Ferulic acid mass spectrum; File S3: p-Coumaric acid mass spectrum; File S4: p-Hydroxybenzoic mass spectrum.

## Sample Availability

Samples of the compounds are not available from the authors.
