# Supplementary figures and images for: Corn Husk Phenolics Modulate Hepatic Antioxidant Response in Nile Tilapia (Oreochromis niloticus) Exposed to Hypoxia
Source: Molecules. 2021 Oct 12;26(20):6161. doi: 10.3390/molecules26206161 (PMC8540891; doi:10.3390/molecules26206161)

F1

Hoja\_090 38 (3.540) Cm (37:38)

2: TOF MSMS 137.02ES-  
3.03e3

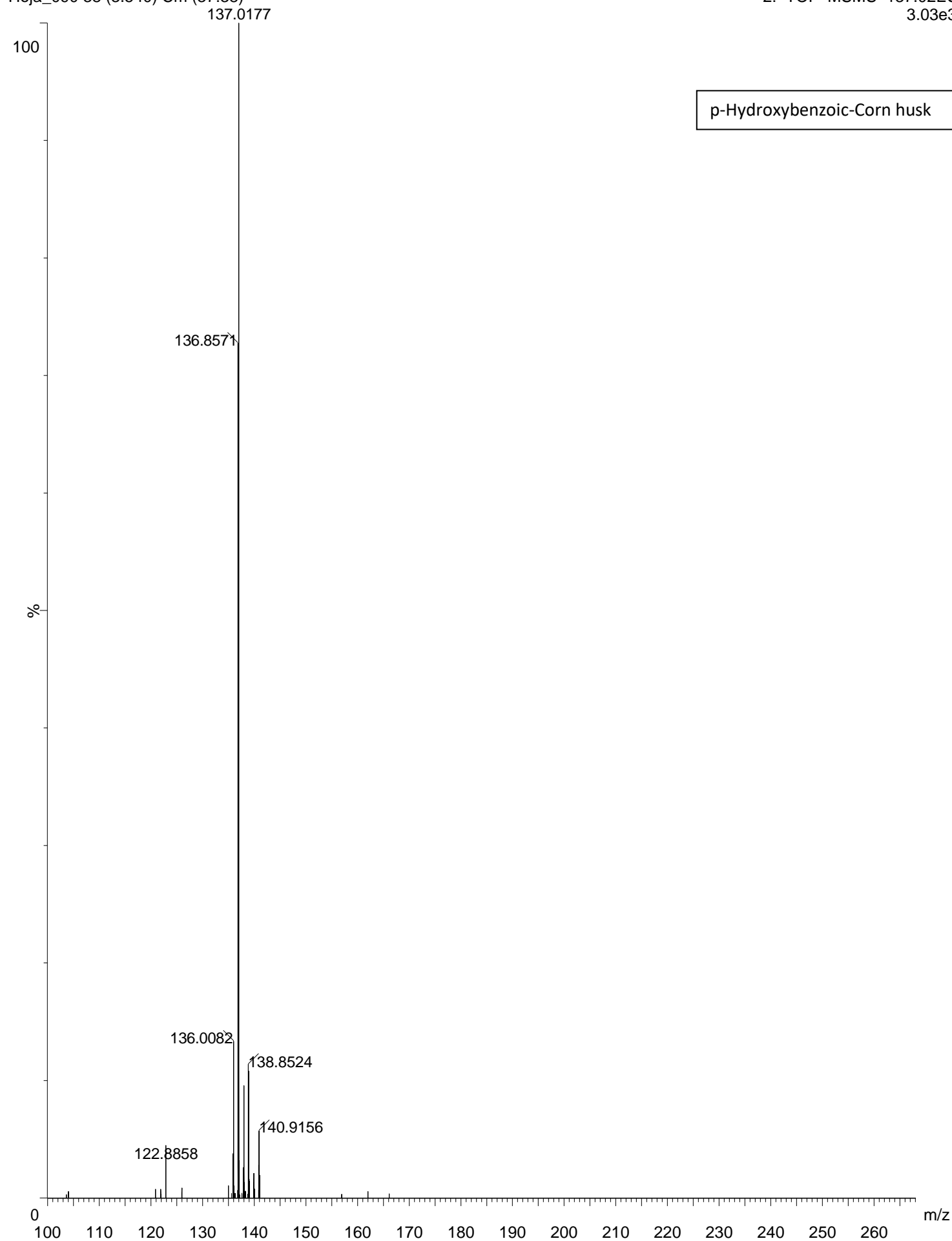

AF 300

Hoja\_079 39 (3.729) Cm (39)

2: TOF MSMS 137.02ES-  
4.60e4

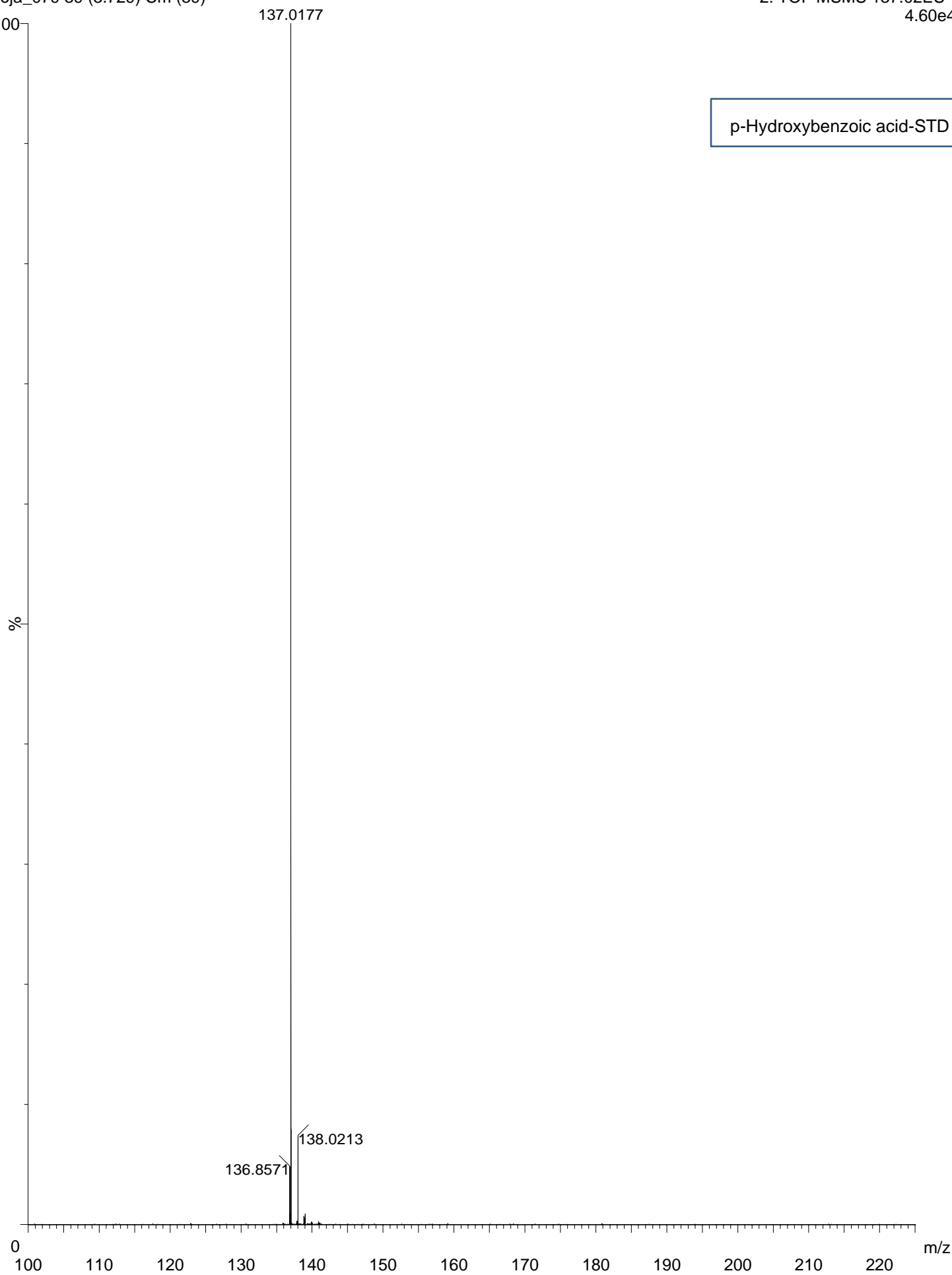

Supplement: Supplementary file 1 [file molecules-26-06161-s001.zip › S4_p Hydroxybenzoic mass spectrum.pdf]

F1

Hoja\_090 26 (3.688) Cm (23:27)

3: TOF MSMS 353.11ES-  
8.08e

5

Chlorogenic acid-Corn husk

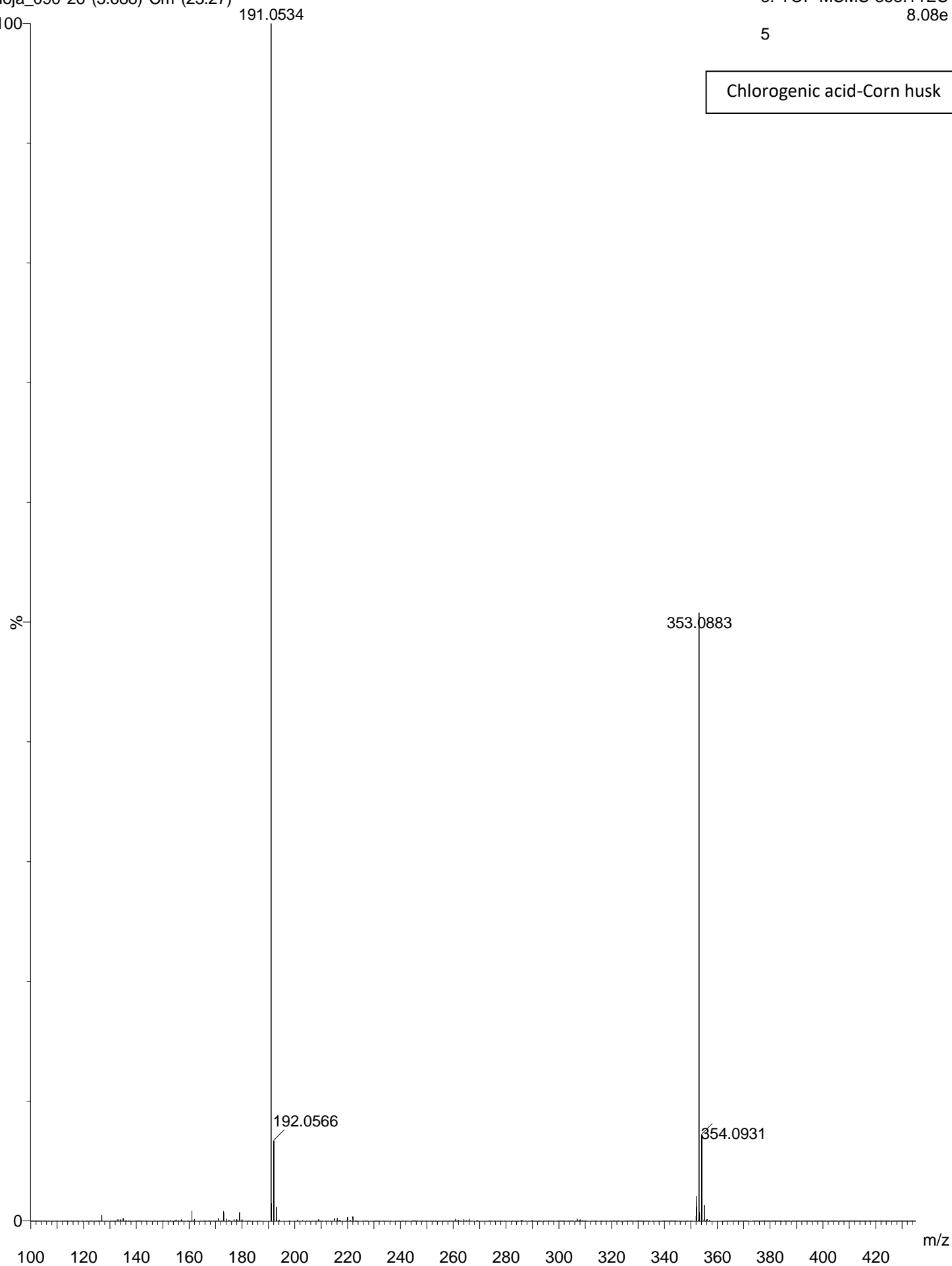

AF 300

Hoja\_079 39 (3.738) Cm (39)  
191.0534

3: TOF MSMS 353.11ES-  
1.54e5

100

Chlorogenic acid-STD

353.0883

192.0566

354.0931

0

160 180 200 220 240 260 280 300 320 340 360 380 400 420 440 460

m/z

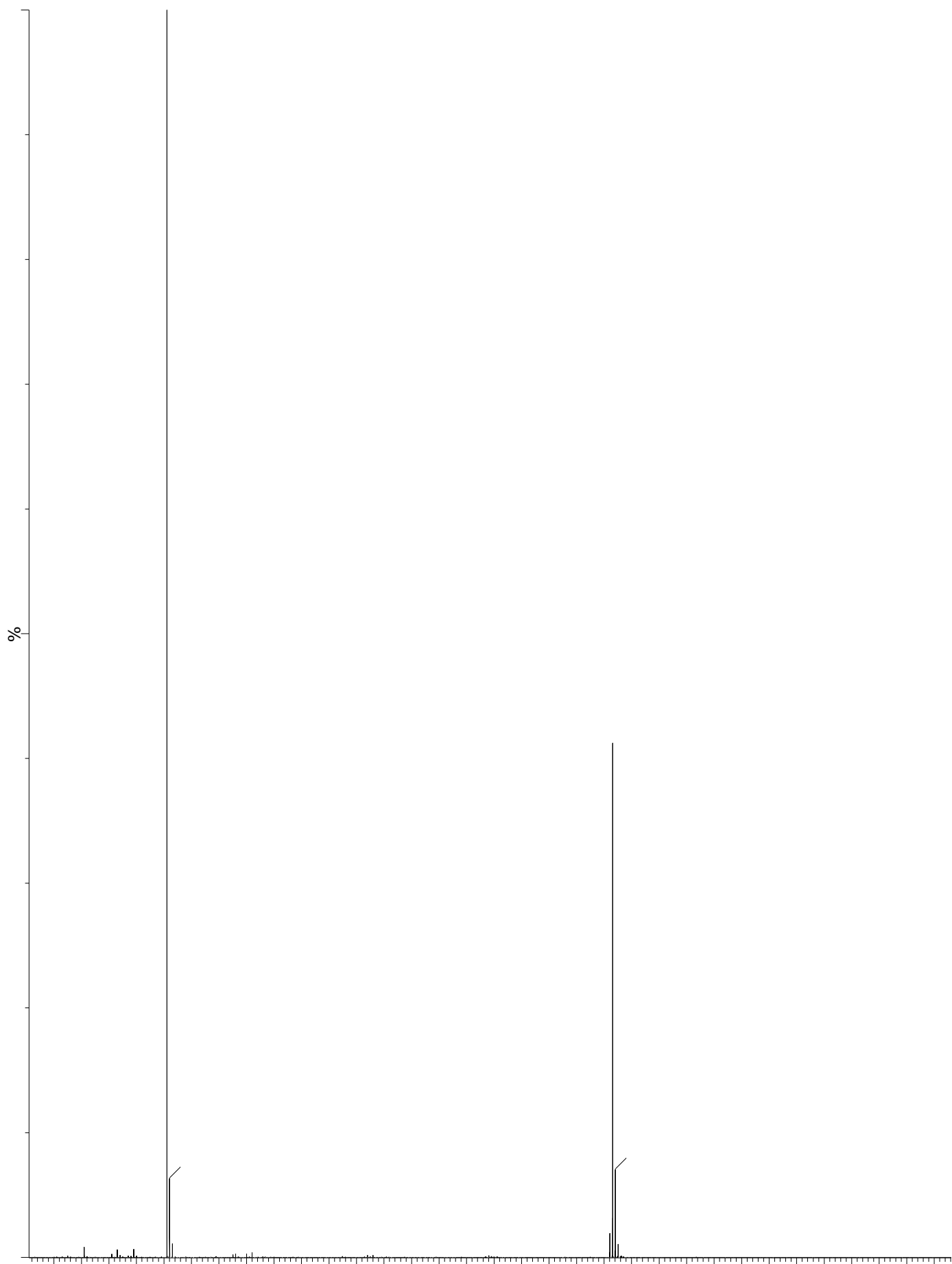

Supplement: Supplementary file 1 [file molecules-26-06161-s001.zip › S1_Chlorogenic acid mass spectrum.pdf]

B1

Hoja\_078 14 (5.455) Cm (14)

9: TOF MSMS 193.05ES-  
5.97e

5

Ferulic acid-Corn husk

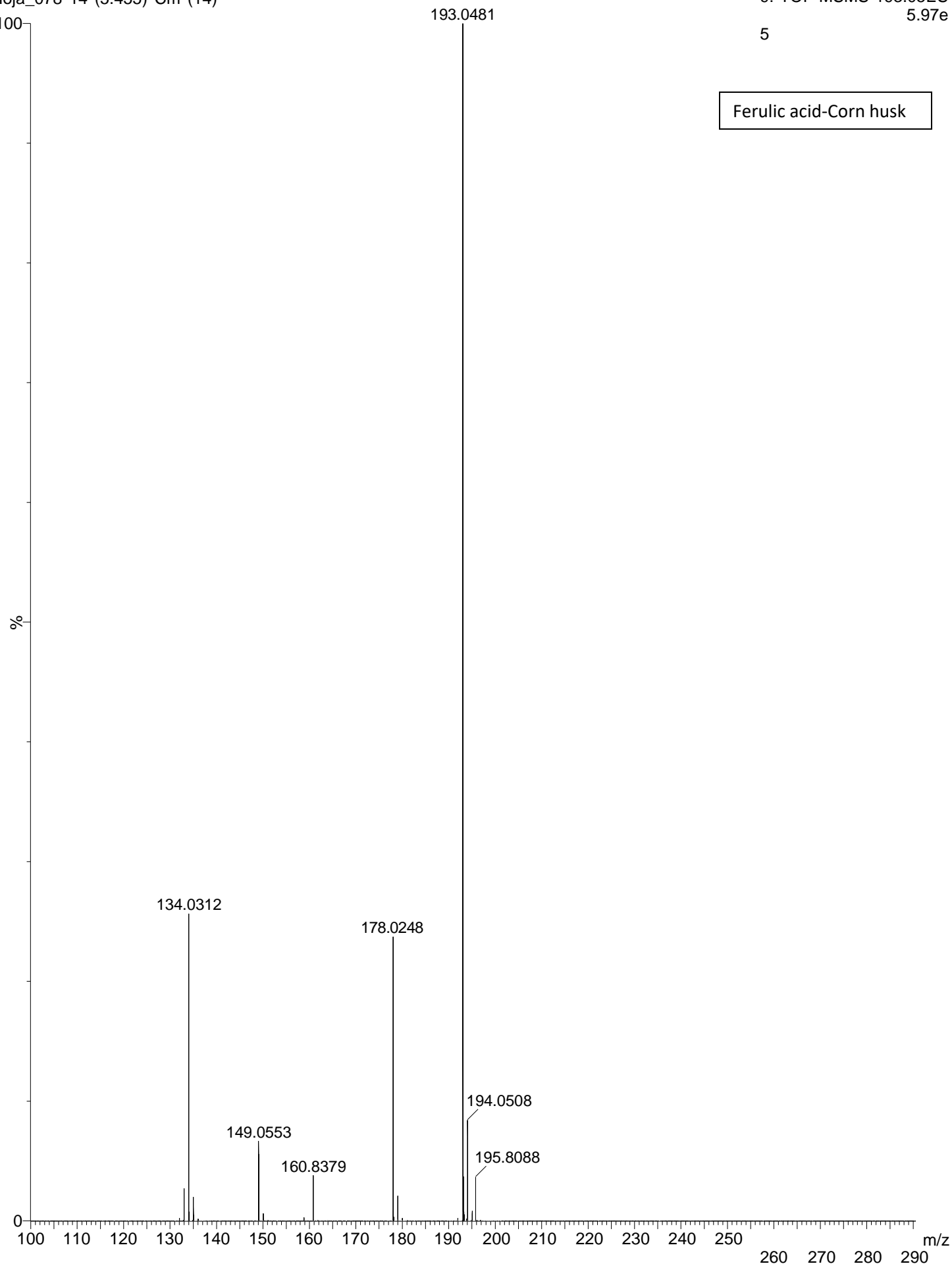

AF 300

Hoja\_079 14 (5.455) Cm (14:15)

9: TOF MSMS 193.05ES-  
7.52e4

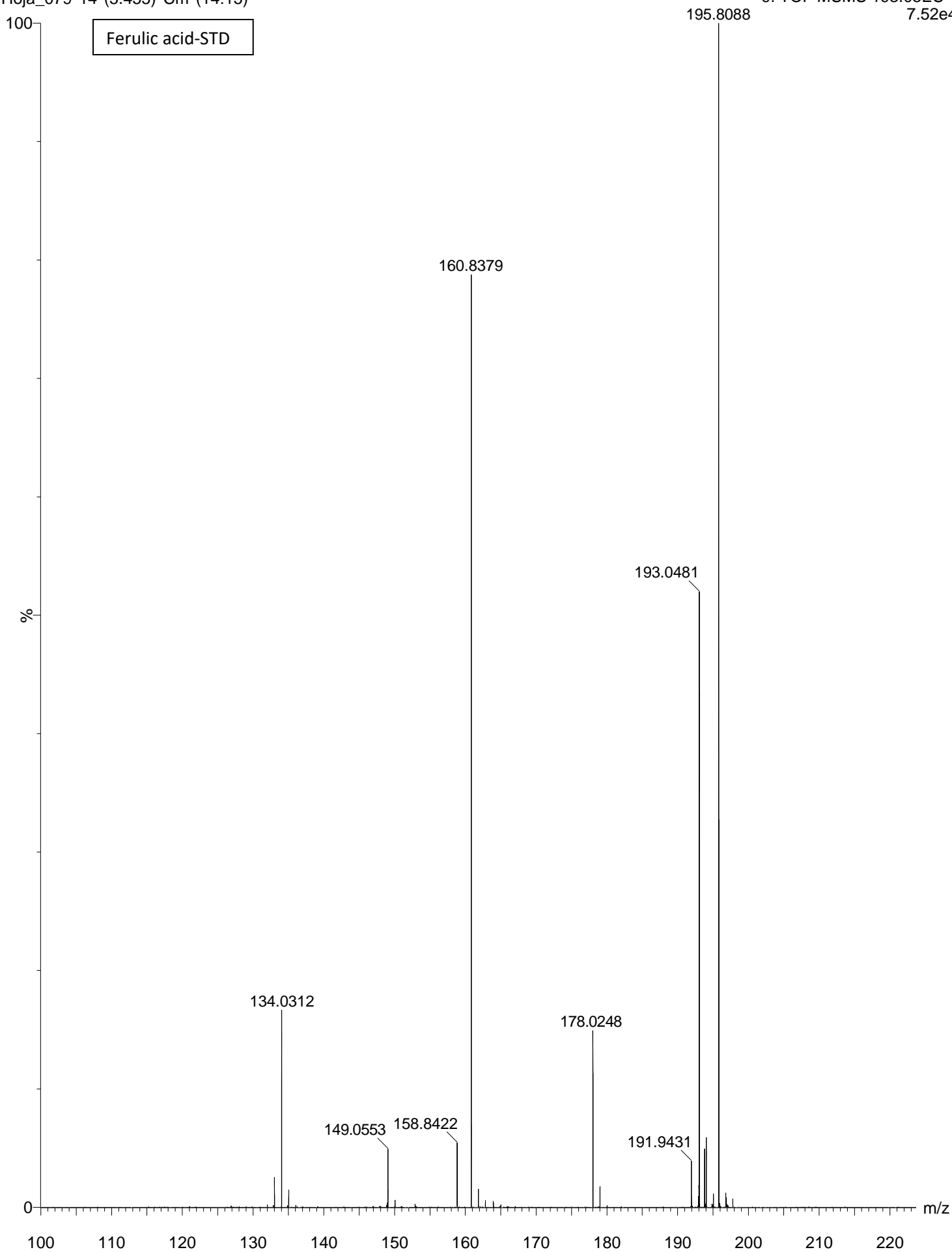

Supplement: Supplementary file 1 [file molecules-26-06161-s001.zip › S2_Ferulic acid mass spectrum.pdf]

F1

Hoja\_090 8 (5.040) Cm (8)

7: TOF MSMS 163.04ES-  
5.36e

4

p-coumaric acid-Corn husk

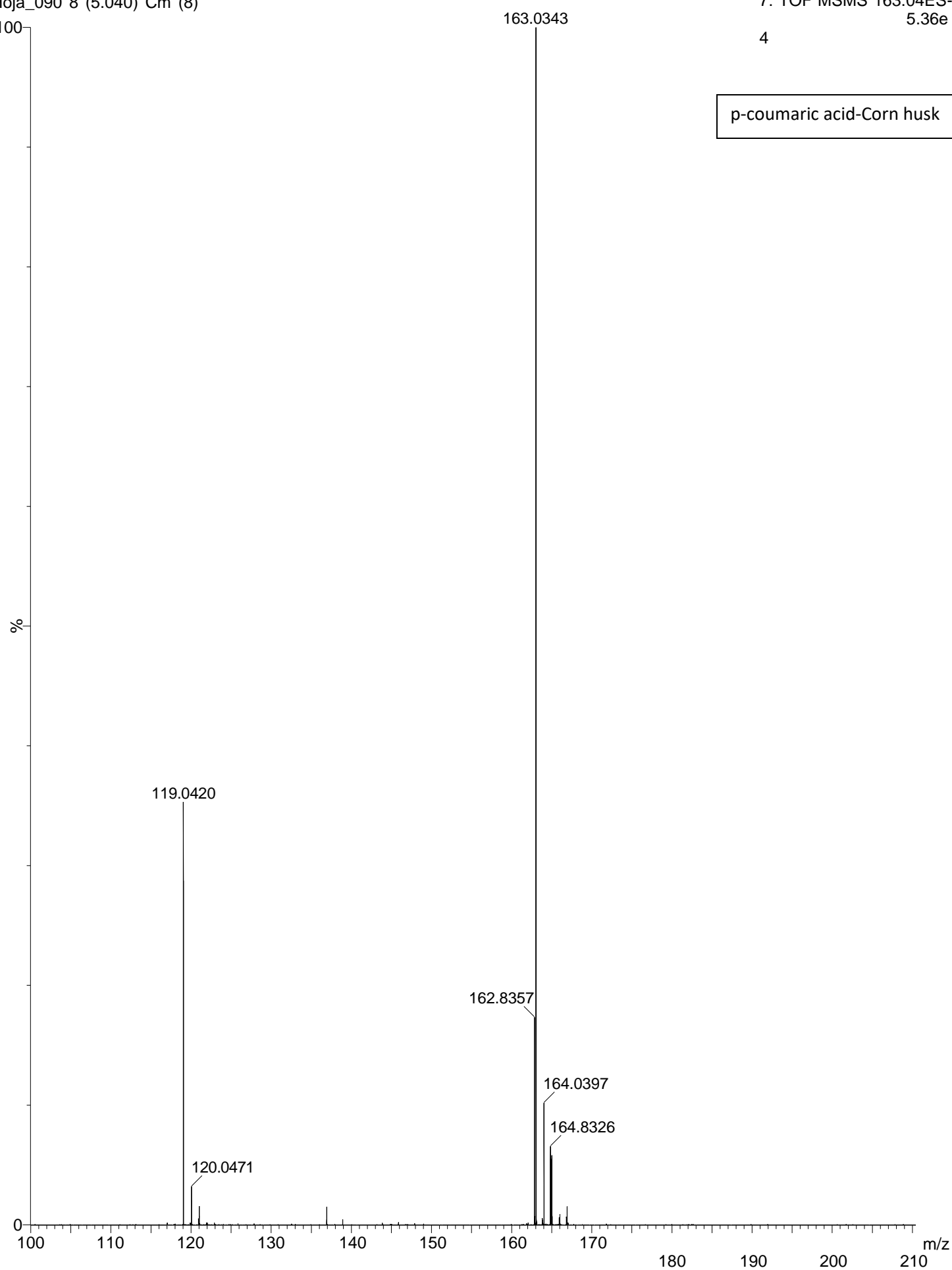

AF 300

Hoja\_079 12 (5.069) Cm (12)

7: TOF MSMS 163.04ES-  
2.19e5

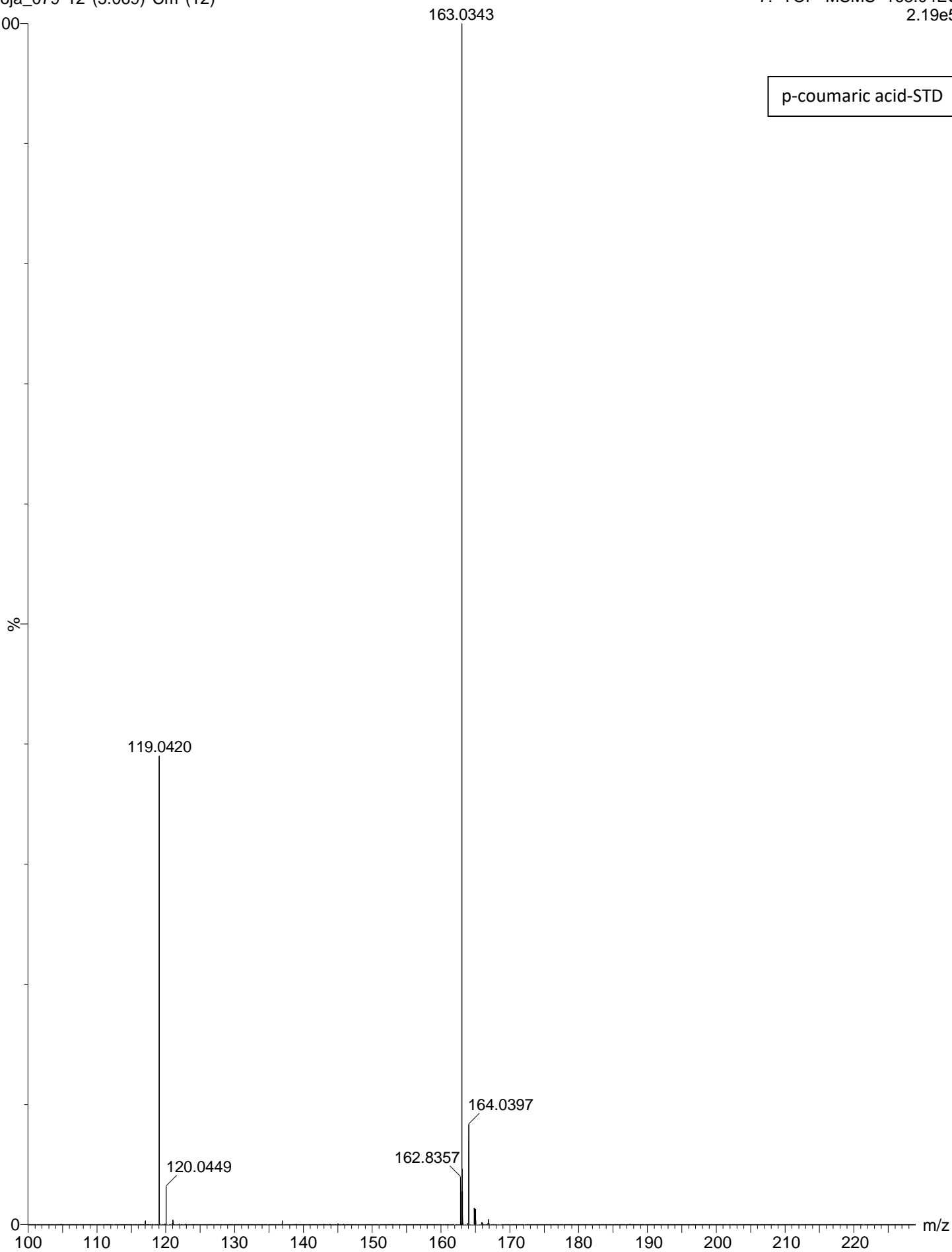

Supplement: Supplementary file 1 [file molecules-26-06161-s001.zip › S3_ p Coumaric acid mass spectrum.pdf]
